# Construction and Activity of Cisplatin-Loaded Chitosan–Zinc Amino-Porphyrin Photosensitizer Hydrogel

**DOI:** 10.3390/gels11120948

**Published:** 2025-11-26

**Authors:** Hongmei Zhang, Dongqing Li, Pengge Wang, Yunxia Yang, Daliang Zhu, Yanqing Wang

**Affiliations:** School of Chemical and Environmental Engineering, Yancheng Teachers University, Yancheng 224007, China; hxzhm@126.com (H.Z.); dqli587@163.com (D.L.); 18736625876@163.com (P.W.); zhudl@yctu.edu.cn (D.Z.)

**Keywords:** photodynamic hydrogel, chitosan, platinum-based drug, drug delivery

## Abstract

Cisplatin resistance remains a major impediment to the successful chemotherapy of various solid tumors, including ovarian, lung, and head and neck cancers. Diverse drug delivery systems with photodynamic specificity significantly target diseased cells precisely. Herein, a homogeneous photodynamic hydrogel drug-loading network based on chitosan (CS) containing zinc amino-porphyrin (ZnTAPP) has been developed for carrying cisplatin (CDDP). Aldehyde groups of glutaraldehyde acted as a bridge to connect ZnTAPP and CS. CDDP was then loaded in CS-ZnTAPP hydrogel to construct the anticancer drug system synergistically. Multiple analysis methods were applied to evaluate the chemical structure and physical properties of hydrogels, including a Fourier transform infrared spectrometer, scanning electron microscopy, an X-ray powder diffractometer, rheological measurements, etc. CS-ZnTAPP hydrogels as well as CS-ZnTAPP-CDDP hydrogels, generated abundant singlet oxygen rapidly for photodynamic therapy. Finally, the hydrogels exhibited significant anticancer activities under irradiation; the IC50 was reduced to 10.936 μg/mL toward CDDP-resistant lung cancer cells (A549/CDDP). The new hydrogel could be applied as a photodynamic anticancer drug delivery system to overcome cisplatin resistance.

## 1. Introduction

Cisplatin is a commonly used chemotherapeutic drug in cancer treatment, but its resistance problem has always been a major clinical challenge. The current landscape of this challenge is characterized by a multifaceted and interconnected network of resistance mechanisms operating within cancer cells. Current research is vigorously focused on identifying predictive biomarkers for resistance, developing novel platinum analogs or combination therapies that target these specific pathways, and exploring epigenetic modulators to re-sensitize resistant cells. Despite these efforts, overcoming cisplatin resistance continues to be a critical and largely unmet clinical need, driving the ongoing search for innovative therapeutic strategies.

In recent years, drug delivery systems based on biomaterials have received widespread attention due to their ability to improve drug targeting and reduce side effects [1,2]. As a kind of biomaterial with unique physical and chemical properties, hydrogel shows great potential in the field of anticancer drug carrier systems [3,4]. Hydrogel is a three-dimensional network structure formed by physical or chemical crosslinking of hydrophilic polymers, which can absorb and maintain a large amount of water while maintaining its structural stability [5]. These unique structures endow hydrogels with the following characteristics: good biocompatibility and degradability, high drug loading and controllable release, multifunctionality, etc. [6,7,8,9]. Chitosan, as a natural polysaccharide from crustacean shells, is widely used in the preparation of anticancer drug hydrogels [10,11]. Based on the advantages of chitosan hydrogel, more and more attention has been paid to some emerging cancer treatment methods, such as photodynamic therapy, immunotherapy, and so on [12]. The construction of cisplatin (CDDP)-loaded chitosan photosensitizer hydrogel is one of the important research directions in this field [13].

**Figure 1:**
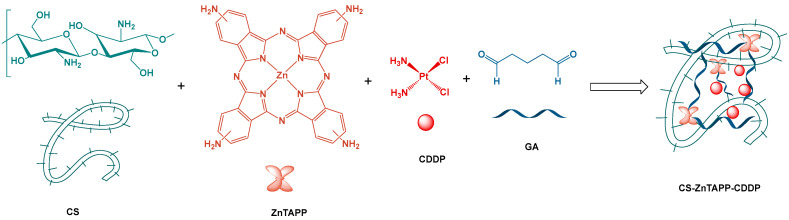
The synthetic routes of CS-ZnTAPP-CDDP hydrogel.

The loaded CDDP hydrogel can realize the local sustained release, stimulus response release, and synergism of combined treatment of CDDP, which has become a research hotspot of tumor drug delivery in recent years [14,15]. In addition, based on the hydrogel platform, photodynamic therapy (PDT) can be combined with tumor microenvironment remodeling and tumor immunotherapy to establish a new and efficient photodynamic immunotherapy model for cancer treatment, which will greatly improve the therapeutic effect of deep solid tumors [16]. Among photosensitizers, porphyrin compounds have excellent photophysical and photochemical properties, such as strong light absorption ability, long excited state lifetime, and efficient singlet oxygen (^1^O_2_) generation ability [17]. Photodynamic therapy possesses the following advantages, such as strong light sensitization ability, targeted modification, and adjustable phototoxicity [18]. The effective assembly of porphyrin photosensitizers within hydrogels solved the problem of poor solubility and expanded their application fields, resulting in improved PDT efficiency [19,20]. In the present study, we developed a facile approach to prepare a photosensitizing hydrogel using chitosan (CS) conjugated with zinc amino-porphyrin (ZnTAPP) with glutaraldehyde (GA) as the linker. GA acted as a bridge to connect chitosan chains and ZnTAPP via a Schiff base reaction between its aldehyde groups and both of the amino groups of chitosan and ZnTAPP. In addition, CDDP was loaded in CS-ZnTAPP hydrogel to construct an anticancer drug system. The preparation process is shown in Figure 1. The new hydrogels will be applied as an ideal photodynamic anticancer drug delivery system and show interesting biological activities.

## 2. Results and Discussion

### 2.1. FTIR Spectral Analysis

**Figure 2:**
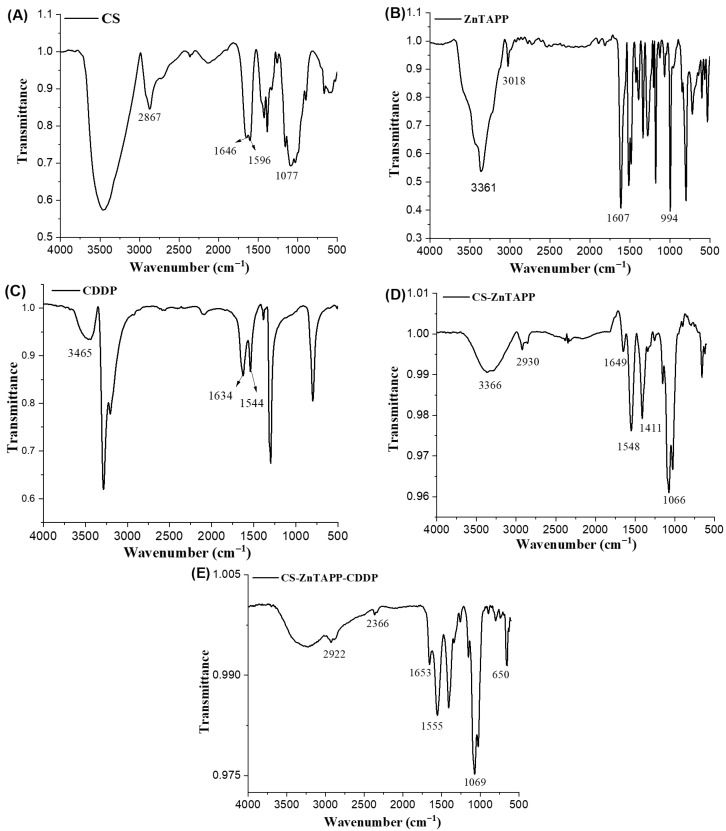
FTIR spectra of CS (**A**), ZnTAPP (**B**), CDDP (**C**), CS-ZnTAPP (**D**), and CS-ZnTAPP-CDDP (**E**).

The FTIR spectrum of CS is shown in Figure 2A; the characteristic absorption peaks of CS were observed at 1077 cm^−1^ and 2867 cm^−1^, corresponding to the C-O and C-H stretching vibrations, respectively. Additionally, two amino peaks appeared at 1646 cm^−1^ and 1596 cm^−1^, which are attributed to the vibrational absorption bands of the amide I and II groups [21]. The FTIR spectrum of ZnTAPP (Figure 2B) showed that the -NH_2_ peaks appear at 3361 cm^−1^ and 3018 cm^−1^, which are attributed to the symmetric and asymmetric stretching vibrations of the -NH_2_ bond, respectively. In the FTIR spectrum of CDDP (Figure 2C), characteristic peaks of the amino group appear at 3465 cm^−1^ and 1634 cm^−1^. In the FTIR spectrum of CS-ZnTAPP, it can be observed that the -CHO group of glutaraldehyde and the -NH_2_ group of ZnTAPP underwent an aldehyde-amine condensation reaction. A new stretching frequency appeared at 1649 cm^−1^, which corresponded to the characteristic stretching vibration peak of the Schiff base C=N double bond. This indicated that chitosan was successfully bonded to ZnTAPP through the Schiff base reaction, confirming the formation of imine bonds in the hydrogel matrix. In the FTIR spectrum of CS-ZnTAPP-CDDP in Figure 2E, the peak near 1555 cm^−1^ decreased in intensity, while the vibrational peak of the functional group near 650 cm^−1^ became sharper, both belonging to the -N-H- stretching vibration. Additionally, by comparing FTIR spectra of CS-ZnTAPP-CDDP, it can be observed that the absorption peak at 3366 cm^−1^ in the CS-ZnTAPP spectrum broadened, and various absorption peaks shifted or disappeared, indicating the additional coordination between CDDP and ZnTAPP and the loading of CDDP within the hydrogel network’s pores.

### 2.2. UV-Vis Spectral Analysis

**Figure 3:**
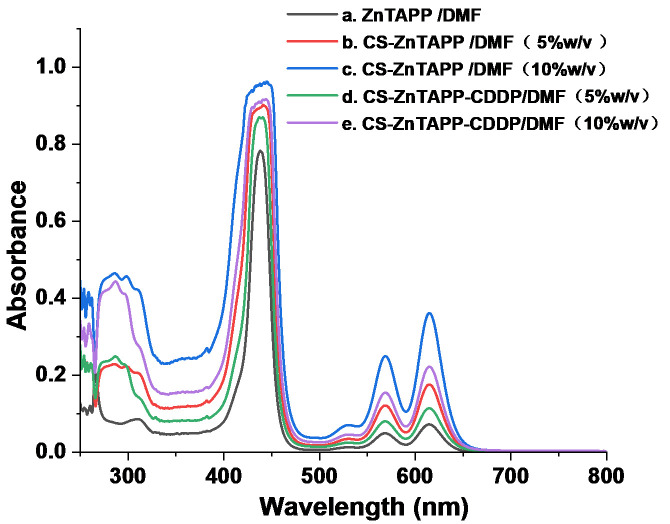
UV-vis spectra of ZnTAPP (a), CS-ZnTAPP (b,c), and CS-ZnTAPP-CDDP (d,e).

As shown in Figure 3a, the UV–Vis spectra of ZnTAPP in DMF consisted of two absorption regions at 300–500 nm and 500–700 nm, which showed the typical spectrum of metalloporphyrin compounds, namely an intense and narrow B (Soret) band and a much weaker Q band [22]. The UV-Vis spectra of CS-ZnTAPP and CS-ZnTAPP-CDDP in DMF with concentrations of 5% and 10% *w*/*v* were shown in Figure 3b–e. As shown in the figure, both CS-ZnTAPP and CS-ZnTAPP-CDDP exhibited the characteristic UV-Vis absorption peaks of ZnTAPP. In addition, the intensity of the characteristic absorption peaks of ZnTAPP in hydrogel increased with concentration, with no new characteristic peaks emerging, indicating the absence of new bands associated with aggregated species.

### 2.3. Pt Content Determination and Analysis

The standard curve of Pt content was obtained according to the ICP-OES experiment (Equation (1)):

y = 4.34 × 10^4^ x − 8.19 × 10^3^ (R^2^ = 0.9966) (1)

where y is the net intensity, and x is the concentration of Pt in standard solutions of different concentrations. Based on Equation (1), the experimental results were obtained as 18.90 mg/L by conducting two parallel experiments. Based on the above results, the percentage of Pt content in CS-ZnTAPP-CDDP hydrogel can be calculated as 3.78%. According to the synthesis procedure of CS-ZnTAPP-CDDP hydrogel, the theoretical Pt content in the CS-ZnTAPP-CDDP hydrogel system is 6.13%, corresponding to an effective CDDP loading efficiency of 61.67%, in fact.

### 2.4. ZnTAPP Content Determination and Analysis

According to the method described in the experiment, the standard curve of ZnTAPP was first plotted to obtain the standard curve equation:

A = 0.2396C + 0.0059 (R^2^ = 0.9936) (2)

where A is the absorbance at 434 nm of ZnTAPP, and C is the concentration of ZnTAPP standard solutions of different concentrations. According to the standard calibration curve, the content of ZnTAPP in CS-ZnTAPP and CS-ZnTAPP-CDDP solutions was calculated as 3.35 and 3.80 mg/L, respectively, which indicated the loading of ZnTAPP within the hydrogel network.

### 2.5. SEM Analysis

**Figure 4:**
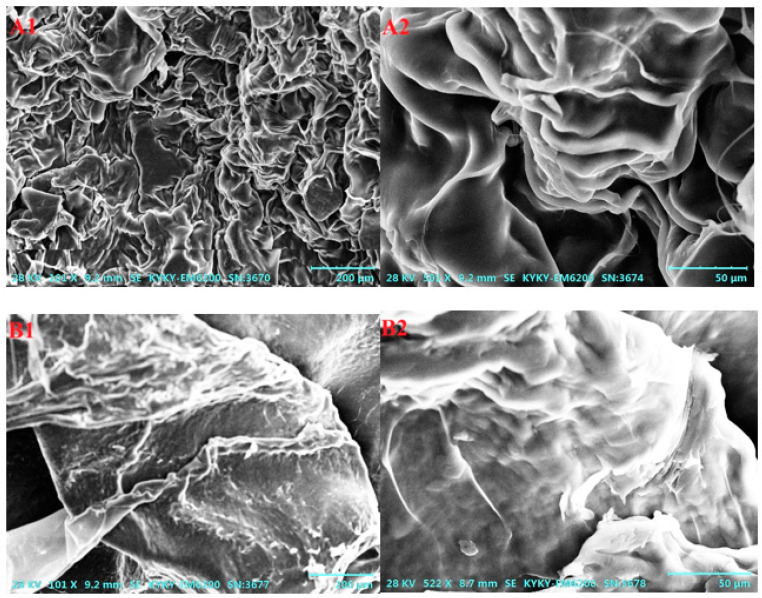
SEM images of CS-ZnTAPP (**A1**,**A2**) and CS-ZnTAPP-CDDP (**B1**,**B2**).

The SEM images of CS-ZnTAPP and CS-ZnTAPP-CDDP are shown in Figure 4. As the results show, the surface of the CS-ZnTAPP hydrogel (Figure 4A1,A2) exhibited an irregular three-dimensional blocky structure, forming a compact structural network. After the incorporation of CDDP, the CS-ZnTAPP-CDDP hydrogel (Figure 4B1,B2) shows no pores, and its surface became rough with numerous nanoscale protrusions, indicating the successful embedding of the small-molecule chemotherapeutic drug cisplatin into the hydrogel network. The observed reduction in pore size and structural densification confirmed the additional crosslinking caused by coordination of CDDP in CS-ZnTAPP hydrogels.

### 2.6. XRD Analysis

**Figure 5:**
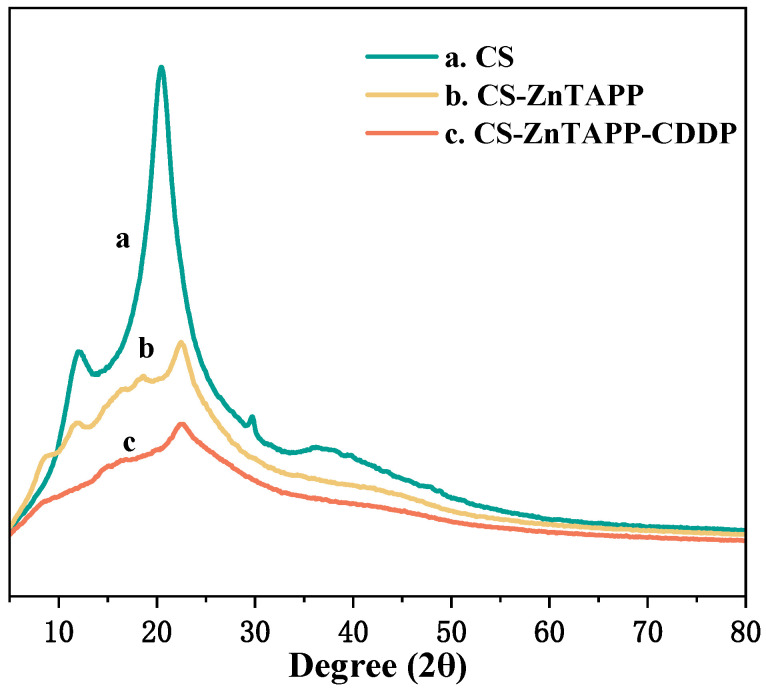
XRD of CS (a), CS-ZnTAPP (b), and CS-ZnTAPP-CDDP (c).

As shown in Figure 5, in the XRD diffraction pattern of CS (Figure 5a), characteristic peaks of CS appeared at 2θ = 12° and 20°, which were attributed to the hydrogen bonding interactions between CS macromolecules [23,24]. These interactions promoted the formation of aggregated networks, resulting in both crystalline and amorphous structures. In the XRD pattern of CS-ZnTAPP (Figure 5b), after modification with ZnTAPP, two characteristic peaks of porphyrin emerged at 2θ = 16° and 18°, though with relatively low intensity, indicating the successful bonding of ZnTAPP to CS. In the XRD pattern of CS-ZnTAPP-CDDP (Figure 5c), the intensity of the characteristic peaks at 2θ = 16° and 18° decreased, and no crystal peak of cisplatin was found in the hydrogel, which strongly indicated that cisplatin has been successfully “amorphized” and integrated into the gel network. The addition of CDDP enhanced the coordination occurrence and maintained the amorphous disordered dispersion package in CS-ZnTAPP hydrogels. The loading of CDDP has a significant impact on the geometric structure of the CS-ZnTAPP hydrogel. This conclusion was in agreement with the results from the SEM experiments.

### 2.7. Rheological Behavior Analysis

**Figure 6:**
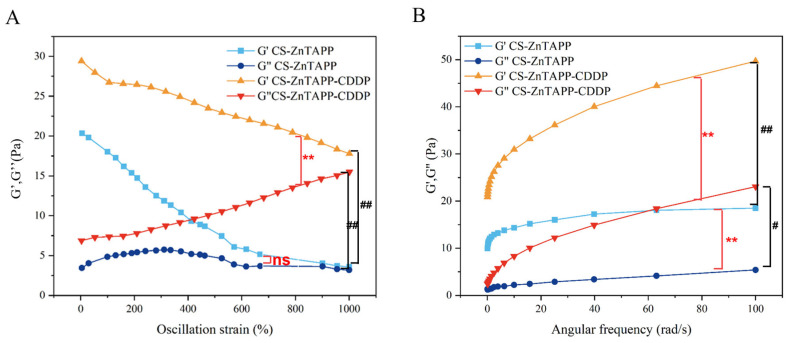
Curves of G′ and G″ of CS-ZnTAPP and CS-ZnTAPP-CDDP hydrogels with dynamic strain changes (**A**) and a function of angular frequency (**B**). All values are expressed as the means ± SEM. Statistical significance was defined as # *p* < 0.05, **## *p* < 0.01 between the two groups. “ns” was defined as “not significant” between the two groups.

Rheological measurements were applied to obtain numerical parameters that define the mechanical properties of the hydrogel, and to evaluate the robustness and structural integrity of its cross-linked network [25,26,27,28]. As shown in Figure 6A, in the curves of storage modulus (G′) and loss modulus (G″) as a function of dynamic strain for CS-ZnTAPP hydrogel and CS-ZnTAPP-CDDP hydrogel at 25 °C, the G′ of CS-ZnTAPP hydrogel decreased sharply within the strain range of 0–570%, and then the decline slowed between 570% and 1000% strain. The G″ of CS-ZnTAPP hydrogel initially increased slowly, began to decrease gradually around 310% strain, and then leveled off after approximately 600% strain. In contrast, the G′ of CS-ZnTAPP-CDDP hydrogel decreased relatively rapidly from 0% to 100% strain and declined uniformly throughout the remaining range. The G″ of CS-ZnTAPP-CDDP hydrogel increased uniformly over the entire dynamic strain range. In both cases, G″ was lower than G′, indicating dominant elastic behavior of CS-ZnTAPP hydrogel and CS-ZnTAPP-CDDP hydrogel. According to Figure 6B, the G′ of both CS-ZnTAPP and CS-ZnTAPP-CDDP hydrogels increased with angular frequency. The G′ of CS-ZnTAPP hydrogel rose sharply at first and then plateaued, while that of CS-ZnTAPP-CDDP hydrogel increased sharply initially and continued to rise steadily. As angular frequency increased, the difference between G′ and G″ was relatively small for CS-ZnTAPP hydrogel, whereas a larger gap was observed for CS-ZnTAPP-CDDP hydrogel, indicating enhanced elasticity in the latter.

Elastic modulus (G′) directly reflects the strength and cross-linking density of the gel network structure. The addition of CDDP increased the elastic modulus significantly in both dynamic rheological tests (Figure 6). Throughout the entire testing range, the G′ of the CS-ZnTAPP-CDDP hydrogels was always higher than that of the CS-ZnTAPP hydrogel, indicating a permanent modulus increase. CDDP interacted with ZnTAPP as well as chitosan chains, adding new, tighter bonding in the hydrogel network and enhanced mechanical properties.

**Figure 7:**
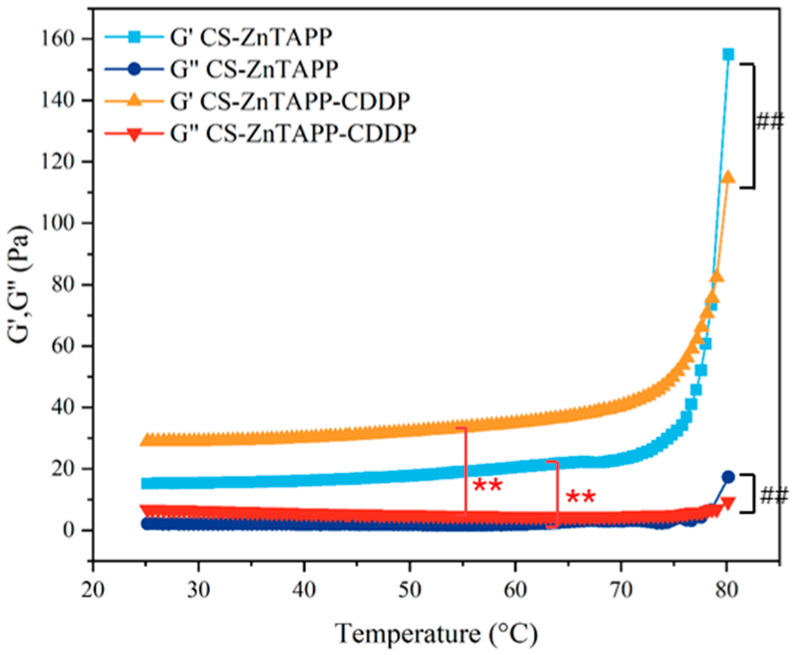
G′ and G″ of CS-ZnTAPP and CS-ZNTAPP-CDDP hydrogels with increasing temperature. All values are expressed as the means ± SEM. Statistical significance was defined as **## *p* < 0.01 between the two groups.

The curves of G′ and G″ of CS-ZnTAPP and CS-ZnTAPP-CDDP hydrogels with increasing temperature are shown in Figure 7. The G″ of both hydrogels showed little change with temperature. The G′ remained relatively stable initially as temperature increased; however, the G′ of CS-ZnTAPP hydrogel increased sharply beyond 75 °C, while that of CS-ZnTAPP-CDDP hydrogel rose more gradually. G′ was much larger than G″ for both hydrogels, demonstrating that elastic behavior predominated throughout the heating process.

To further explore the phenomenon of increased G′, the CS-ZnTAPP hydrogels were synthesized at different temperatures to form the internal network (50 and 80 °C). The free amino groups were measured, and the results are shown in Figure S2. The absorbance value of the hydrogels maintained at 80 °C was significantly reduced, indicating that more amino groups reacted as the Schiff base formed. Consistent with our results, the crosslinking reaction was enhanced with increased temperature beyond 75 °C.

### 2.8. Degradation Properties of Hydrogels

**Figure 8:**
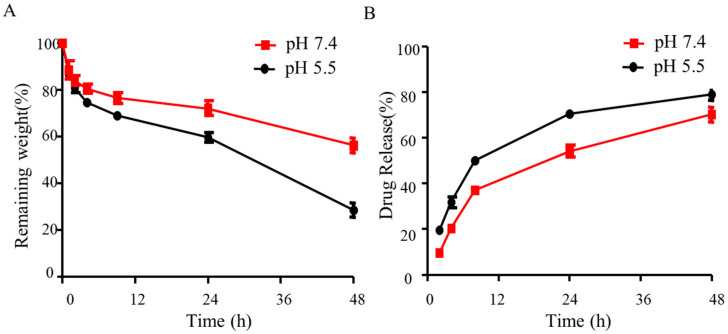
The degradation properties (**A**) and drug release profile (**B**) of hydrogels under different pH conditions (pH 7.4 and 5.5).

To visualize the stability of hydrogels, the degradation rate of hydrogels was monitored by examining weight loss at each time interval. Lysozyme was added to simulate body fluids and promote degradation in vivo [29]. As shown in Figure 8A, CS-ZnTAPP-CDDP hydrogels were degraded to 60% of their remaining weight at pH 7.4. The degradation rate was promoted at pH 5.5, and the remaining weight was reduced to almost 30% over 48 h. The results were attributed to the cleavage of Schiff’s base in an acidic environment. This was consistent with the tendency observed in the case of succinoglycan dialdehyde-crosslinked alginate hydrogels [30].

### 2.9. Drug Release of Hydrogels

The drug release experiment of CDDP was performed via a dialysis method, and the results were displayed in Figure 8B. Cisplatin was found to be released cumulatively with a 60% release rate within 48 h, confirming the local sustained release function. The release rate was enhanced in an acidic environment (pH 5.5). Such a pH-responsive drug release behavior should be attributed to the instability of Schiff’s base in an acidic environment [31]. An imine bond undergoes accelerated hydrolysis in acidic conditions but exhibits relative stability at physiological pH [32]. For this reason, the CS-ZnTAPP-CDDP hydrogels exhibited accelerated degradation and drug release in acidic conditions.

### 2.10. Singlet Oxygen (^1^O_2_) Production of Hydrogels

**Figure 9:**
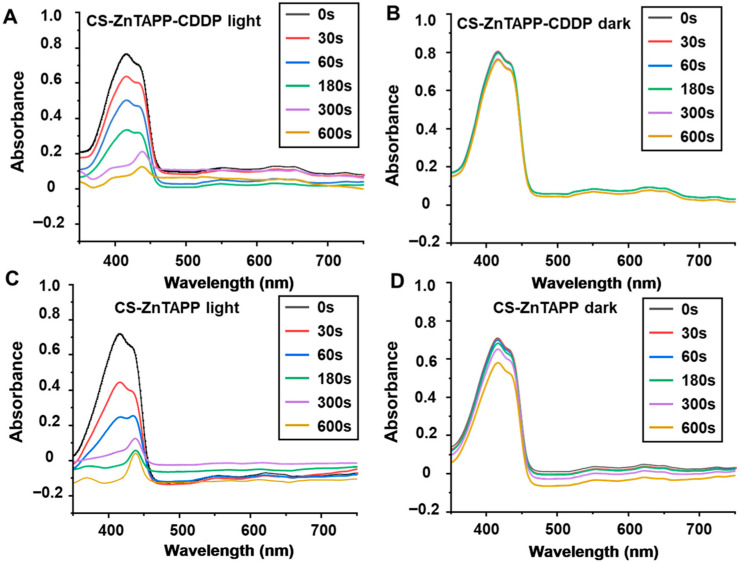
UV-Vis of singlet oxygen production of hydrogels under NIR irradiation over a 10-minute time period. (**A**) CS-ZnTAPP-CDDP hydrogel under light at 100 mW/cm^2^; (**B**) CS-ZnTAPP-CDDP hydrogel under dark conditions; (**C**) CS-ZnTAPP hydrogel under light at 100 mW/cm^2^; (**D**) CS-ZnTAPP hydrogel under dark conditions.

The singlet oxygen (^1^O_2_) production of CS-ZnTAPP-CDDP and CS-ZnTAPP hydrogels was determined in Figure 9. As shown in Figure 9, DPBF exhibits a characteristic absorption peak at 417 nm, and the hydrogels with DPBF exhibited two typical absorption peaks, reflecting DPBF and ZnTAPP at 417 nm and 434 nm, respectively. CS-ZnTAPP-CDDP as well as CS-ZnTAPP hydrogels exhibited rapid photodegradation of DPBF within 600 s under light irradiation (Figure 9A,C), confirming the superior PDT efficiency of both hydrogels. The two hydrogels in dark conditions showed weak abilities to generate singlet oxygen (Figure 9B,D). Moreover, the DPBF reduction was qualified in Figure S2; CS-ZnTAPP hydrogels exhibited a faster ^1^O_2_ generation capability compared with CS-ZnTAPP-CDDP hydrogels. This phenomenon was due to the closer coordination between ZnTAPP and CDDP, confirmed by XRD, SEM, and rheology analysis. This cross-molecular coordination bond reduced the ^1^O_2_ generation capability as a result. Consistent with the reported paper, the drug release rates and singlet oxygen generation will be modulated by tuning the cross-linking degree of related hydrogels [33].

### 2.11. Anticancer Activity of Hydrogels

**Figure 10:**
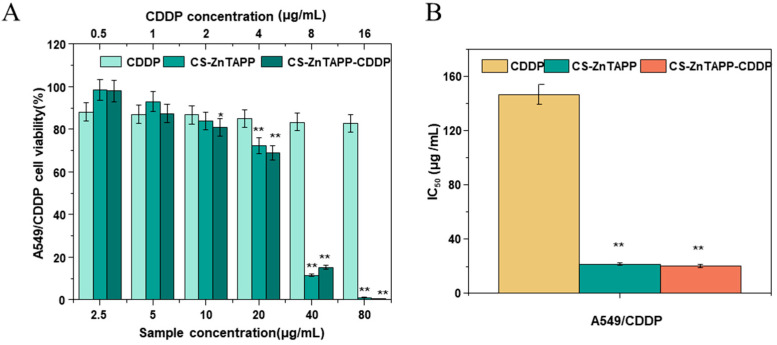
Cell survival curves of A549/CDDP after 48 h (**A**) and IC50 values of CDDP, CS-ZnTAPP, and CS-ZnTAPP-CDDP (**B**). All values are expressed as the means ± SEM. Statistical significance was defined as * *p* < 0.05, ** *p* < 0.01 compared with the CDDP group.

In this work, the in vitro cytotoxicity of the CS-ZnTAPP and CS-ZnTAPP-CDDP hydrogels was evaluated in A549/CDDP cells using the MTT assay. The chitosan and ZnTAPP alone were not investigated in the study due to previous studies [34,35]. Chitosan has been widely applied as a drug carrier and exhibited no cytotoxicity on cells [35]. The porphyrin and its derivatives are nontoxic to the lung cancer cell line A549 in the dark [34]. The cell viability curves of A549/CDDP treatment with CDDP, CS-ZnTAPP, and CS-ZnTAPP-CDDP, and the IC50 data for CDDP, CS-ZnTAPP, and CS-ZnTAPP-CDDP are shown in Figure 9A and 9B. As shown in Figure 10A, after 48 h of co-culture with the cells, CDDP, CS-ZnTAPP, and CS-ZnTAPP-CDDP exhibited a certain concentration-dependent inhibitory effect on cell proliferation. The IC50 values for CDDP, CS-ZnTAPP, and CS-ZnTAPP-CDDP were 146.739 μg/mL, 21.573 μg/mL, and 20.291 μg/mL, respectively (Figure 10B). CS-ZnTAPP and CS-ZnTAPP-CDDP demonstrated significant toxicity toward A549/CDDP cells. These results indicated that the combined effect of CDDP and ZnTAPP enhanced the efficacy against CDDP-resistant lung cancer cells.

**Figure 11:**
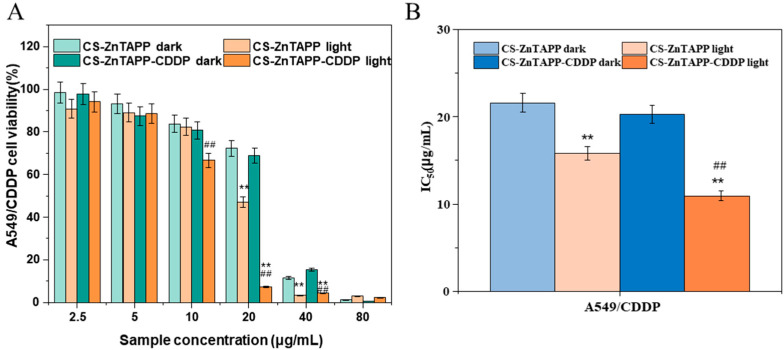
Cell survival curves of A549/CDDP after 48 h (**A**) and the IC50 values of CS-ZnTAPP and CS-ZnTAPP-CDDP under dark and light conditions (**B**). All values are expressed as the means ± SEM. Statistical significance was defined as ** *p* < 0.01 compared with the corresponding dark group; ## *p* < 0.01 compared with the CS-ZnTAPP light group.

Herein, the photodynamic cytotoxicity of the CS-ZnTAPP and CS-ZnTAPP-CDDP hydrogels was also evaluated in A549/CDDP cells using the MTT assay. The cell viability curves of A549/CDDP under dark and light conditions and the IC50 data for CDDP, CS-ZnTAPP, and CS-ZnTAPP-CDDP hydrogels are shown in Figure 11. The data showed no cytotoxic effects of the light irradiation alone (Figure S3), consistent with the reported results [36,37]. As shown in Figure 10, after 48 h of culture with A549/CDDP cells, CS-ZnTAPP and CS-ZnTAPP-CDDP exhibited a certain dose-dependent inhibitory effect on the proliferation of both cell types. Under 420–430 nm light irradiation, the cytotoxicity significantly increased, with IC50 values for CS-ZnTAPP and CS-ZnTAPP-CDDP of 15.788 μg/mL and 10.936 μg/mL, respectively. In conclusion, CS-ZnTAPP and CS-ZnTAPP-CDDP demonstrated notable phototoxicity toward A549/CDDP cells. The efficacy of this system may be limited to a single specific cell line, and further verification studies across a broader range of normal cells and more cancer cell lines (e.g., ovarian cancer, head and neck cancer) are required to confirm its potential as a universal resistance-overcoming therapeutic.

## 3. Conclusions

The research on cisplatin resistance in cancer treatment is constantly advancing. In the study, a new hydrogel, CS-ZnTAPP-CDDP, was developed for the treatment of cisplatin resistance using a photosensitive strategy. The hydrogel was synthesized via a cross-linking process between ZnTAPP and CS rapidly. CS-ZnTAPP and CS-ZnTAPP-CDDP hydrogels exhibited notable phototoxicity, and the IC50 was reduced to 10.936 μg/mL toward A549/CDDP cells. Further studies on other cisplatin-resistant cancers, in vivo efficacy, drug release kinetics, systemic toxicity, or long-term effects are crucial for future applications. The novel hydrogels were expected to provide more effective treatment options for cancer patients in the future.

## 4. Materials and Methods

### 4.1. Materials

CS (pharmaceutical grade, degree of deacetylation (%), ~95%) was purchased from Qingdao Bozhihuili Biotechnology Co., Ltd. (Qingdao, China), CDDP (~99%) was purchased from Jinan Renyuan Chemical Co., Ltd. (Jinan, China) RES, ZnTAPP (~98%), was purchased from Jilin Zhongke Extension Technology Co., Ltd. (Changchun, China). The A549/CDDP cell line (cisplatin-resistant human non-small cell lung cancer cell line) was purchased from the American Type Culture Collection. All other chemicals were analytical grade and used as received.

### 4.2. Synthesis of CS-ZnTAPP-CDDP Hydrogel

The construction method of the hydrogel network was modified based on the relevant literature [38]. First, the acetic acid solution was diluted to 1% (*v*/*v*), and the 25% glutaraldehyde aqueous solution was diluted to 1% (*v*/*v*). Next, 0.15 g of CS was weighed and added to 1% (*v*/*v*) acetic acid aqueous solution (5 mL). The mixture was stirred in a 50 °C water bath for 15 min to ensure complete dissolution. Subsequently, CDDP (10 mg) was added to the mixture, followed by heating and stirring for 10 min. Then, 3 mg of ZnTAPP was weighed and dissolved in 1 mL of DMF solution. After thorough dissolution, the solution was added dropwise to the mixture using a pipette. The mixture was returned to the water bath and stirred for an additional 5 min. After cooling to room temperature, 300 μL of the diluted glutaraldehyde solution was added dropwise. After preparing the homogeneous solution for 10 min, the mixture was heated again to 50 °C. After 6 min, it was cooled back to room temperature to form the hydrogel network. Glutaraldehyde acted as a bridge to connect chitosan chains and ZnTAPP via Schiff base reaction. Subsequently, the prepared hydrogel was immersed in distilled water (5 mL) to remove unreacted chemicals such as DMF. Finally, the hydrogel matrix was rinsed with excess distilled water (10 mL) to eliminate any unreacted substances on the surface of the hydrogel.

### 4.3. Characterization of Hydrogels

A UV/Vis spectrophotometer (LAMBDA25, PerkinElmer Corporation, Waltham, MA, USA) was used to record the spectra of the sample with a wavelength range from 300 nm to 800 nm. A VERTEX 80/Raman II Fourier transform infrared spectroscopy (FTIR) spectrometer (Bruker, Billerica, MA, USA) was used to obtain the FTIR spectra of the sample with the range from 500 to 4000 cm^−1^. The prepared hydrogels were quickly frozen in liquid nitrogen and then lyophilized for 48 h [39]. The surface morphology of freeze-dried hydrogels was observed after coating with a thin layer of gold using a Scanning Electron Microscope (KYKY-EM6200/6900 model) with an acceleration voltage of 15 kV. The samples were tested on an X-ray powder diffractometer (Ultima IV, Rigaku Corporation, Tokyo, Japan) with a diffraction angle of 5–80° and a scanning speed of 5 °/min.

### 4.4. Pt Content Measurement

The platinum standard solution with a concentration of 100 mg/L was diluted with 10% hydrochloric acid to obtain platinum standard solutions with concentrations of 2 mg/L, 4 mg/L, 6 mg/L, 8 mg/L, and 10 mg/L. The calibration curve for the platinum standard solutions was then determined using an Inductively Coupled Plasma Optical Emission Spectrometer (ICP-OES) (OPTIMA8000DV) (PerkinElmer, Waltham, MA, USA) in order to measure the Pt content of CS-ZnTAPP-CDDP hydrogel.

Accurately weigh two 5 mg samples of CS-ZnTAPP-CDDP hydrogel, moisten each with 1 mL of ultrapure water, add 2 mL of aqua regia, and perform nitrification in an oil bath at 125 °C. Repeat this process multiple times until the hydrogel samples are completely dissolved. Filter the solution through a 0.45 μm disposable needle filter, transfer the filtered clear solution into a 10.00 mL brown volumetric flask, and bring it to volume. Finally, determine the platinum content in the CS-ZnTAPP-CDDP hydrogel using ICP-OES according to the platinum calibration curve.

### 4.5. ZnTAPP Content Measurement

The ZnTAPP solution with a concentration of 1 mg/mL was prepared by dissolving 5 mg of ZnTAPP in 5 mL of DMF, which was then diluted to different concentrations. A standard calibration curve of ZnTAPP was plotted with the mass concentration of ZnTAPP as the x-axis and the absorbance at 434 nm as the y-axis. The CS-ZnTAPP-CDDP (100 mg) was dissolved in 10 mL of DMF and stirred magnetically at room temperature for 48 h to ensure complete dissolution. The solution was then diluted to obtain a sample concentration of 120 mg/L. Subsequently, the absorbance at 434 nm was measured to determine the content of ZnTAPP in CS-ZnTAPP-CDDP according to a standard calibration curve.

### 4.6. Rheological Performance Testing

DHR-2 (TA Company, Boston, MA, USA) was used to analyze the rheological performance of hydrogels. The test employed 40 mm parallel plate aluminum fixtures with a plate spacing set at 1 mm. A plastic dropper was cut short to facilitate the aspiration of hydrogels. Using the dropper, each of the hydrogels was evenly spread on the testing plate in a circular shape. During the sample loading process, gentle and slow movements were required to prevent gaps in the hydrogels, ensuring that the sample filled the space between the fixture and the plate once the set spacing was achieved. Excess sample at the edges of the fixture was then removed using a thin plastic sheet. In the test measuring storage modulus (G′) and loss modulus (G″) as a function of strain % at a constant frequency, the temperature was 25 °C, the frequency was fixed at 1 Hz, and the strain ranged from 0.01% to 1000%. For the temperature sweep test, the temperature was first increased from 25 °C to 80 °C at a heating rate of 5 °C/min. Throughout the temperature sweep experiment, a frequency of 1 Hz and a strain of 1% were applied. In the test measuring G′ and G″ as a function of angular frequency (ω) at a strain of 1%, the temperature was maintained at 25 °C, and ω was varied over a range of 0.1 rad/s to 100 rad/s.

### 4.7. Measurement of Free Amino Groups

The CS-ZnTAPP hydrogels were synthesized at different temperatures to form the internal network (50 and 80 °C). The obtained hydrogels were lyophilized, and the residual amino groups were measured according to the research previously [26]. The hydrogels (0.5 mg/mL) were resolved in water overnight before boiling with the ninhydrin solution (2.0%, *w*/*w*) in the dark for 20 min. The OD value was measured at 570 nm after cooling for 20 min.

### 4.8. Degradation Determination of Hydrogels

The degradation determination of hydrogels was performed with minor modifications according to previous studies [30]. Hydrogels were weighed (w0) and immersed in PBS buffer (pH = 7.4, 5.5) solution with 1 mg/mL lysozyme [29]. The hydrogels were taken out and weighed again (wt) over a period of time. The remaining solution on the surface was carefully removed before measurements. The remaining weight was determined using the equation below: Remaining weight (%) = wt/w0 × 100.

### 4.9. In Vitro CDDP Release

A dialysis method was used to investigate drug release profiles of hydrogels. Briefly, hydrogels were placed in 3500 Da MWCO dialysis bags containing PBS solution with 1 mg/mL lysozyme (pH 7.4 and pH 5.5) [29,40]. The dialysis tubes were kept in a constant-temperature shaking table (150 rpm, 37 °C). 5.0 mL of dialysis solution was periodically taken out for the ICP-OES measurement of the amount of Pt, and an equal volume of fresh medium was added.

### 4.10. Singlet Oxygen Generation

Singlet oxygen generation was quantified using 1,3-diphenylisobenzofuran (DPBF, Merck, Darmstadt, Germany) with a concentration of 1 × 10^−2^ mM in DMF. The CS-ZnTAPP-CDDP and CS-ZnTAPP hydrogels were dissolved in DMF (0.5%, *w*/*w*), and the reduction in DPBF concentration was determined at 417 nm over a 10-minute time period with or without light irradiation (420–430 nm, 100 mW/cm^2^). DPBF acts as a chemical quencher and weakens rapidly when singlet oxygen reacts with it. The DPBF absorbance values against irradiation time indicate the photosensitizer’s potency to produce singlet oxygen.

### 4.11. Cytotoxicity Studies

This study utilized A549/CDDP (cisplatin-resistant human non-small cell lung cancer cell line) and employed the MTT [3-(4,5-dimethyl-2-thiazolyl)-2,5-diphenyl-2H-tetrazolium bromide method] following the experimental procedures outlined in the MTT Cell Proliferation and Cytotoxicity Assay Kit manual [41]. The specific experimental steps were as follows.

A549/CDDP cells were cultured in 1640 medium supplemented with 10% (*v*/*v*) fetal bovine serum (FBS) under humidified air containing 5% CO_2_ at 37 °C. For the assay, 100 μL of cell suspension (approximately 6000–8000 cells per well) was seeded into a 96-well plate and allowed to adhere for 24 h. After incubation, 100 μL of sample solution (with a concentration gradient of 0–160 μg/mL) was added to each well. The cells were then cultured for an additional 48 h. Following drug treatment, 50 μL of MTT solution was added to each well and incubated for 4 h to allow formazan (blue-purple precipitate) formation. The supernatant was carefully removed, and 150 μL of DMSO was added to dissolve the formazan crystals. The absorbance at 570 nm was measured using a microplate reader. and each experiment was performed in triplicate.

To investigate the cytotoxicity of CS-ZnTAPP and CS-ZnTAPP-CDDP against A549/CDDP cells under dark and light conditions, the following procedure was conducted: A 100 μL cell suspension (containing A549/CDDP cells) was seeded into each well of a 96-well plate and incubated for 24 h to allow cell adhesion. Subsequently, 100 μL of sample solution (with a concentration gradient of 0–160 μg/mL) was added to each well. After 4 h of drug treatment, the dark groups were shielded from light throughout the entire incubation period, and the light groups were exposed to 420–430 nm light for 20 min, followed by continued incubation for an additional 44 h in the dark. The exact energy dose of the light source was 100 mW/cm^2^, and the distance was 2 cm away from the cell plates [42]. Control cells (0 μg/mL without hydrogels) were cultured for 72 h in the laboratory, and the percentage of viable cells was calculated relative to the untreated control group. Finally, the MTT assay was performed as described previously to assess cell viability.

### 4.12. Statistical Analysis

Two-way ANOVA was performed for statistical analysis. All values are expressed as the means ± standard error of mean (SEM). Statistical significance was defined as * *p* < 0.05, ** *p* < 0.01, and *** *p* < 0.001.

## Data Availability

The original contributions presented in this study are included in the article. Further inquiries can be directed to the corresponding authors.

## Supplementary Materials

The following supporting information can be downloaded at: https://www.mdpi.com/article/10.3390/gels11120948/s1, Figure S1: The measurement of free amino groups in CS-ZnTAPP-CDDP hydrogels reacted in different temperatures; Figure S2: Single oxygen-induced consumption of DPBF of hydrogels over a 10-min time period; Figure S3: Cell survival curves of A549/CDDP after 48 h under dark and light conditions alone.
