# Supplementary material for: Construction and Activity of Cisplatin-Loaded Chitosan–Zinc Amino-Porphyrin Photosensitizer Hydrogel
Source: Gels. 2025 Nov 26;11(12):948. doi: 10.3390/gels11120948 (PMC12732534; doi:10.3390/gels11120948)
Supplement: Supplementary file 1 [file gels-11-00948-s001.zip › gels-3954192-supplementary.pdf]

**Construction and Activity of Cisplatin-Loaded Chitosan-Zinc Amino-porphyrin Photosensitizer Hydrogel**

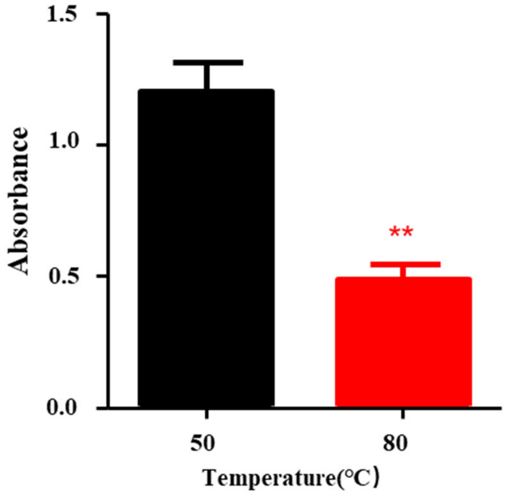


**Figure S1.** The measurement of free amino groups in CS-ZnTAPP-CDDP hydrogels reacted in different temperatures.

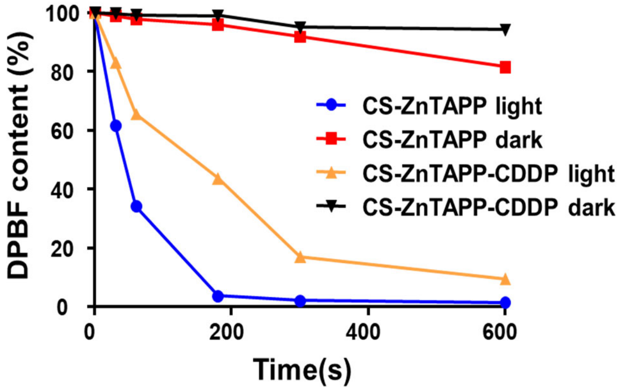


**Figure S2.** Single oxygen-induced consumption of DPBF of hydrogels over a 10-min time period.

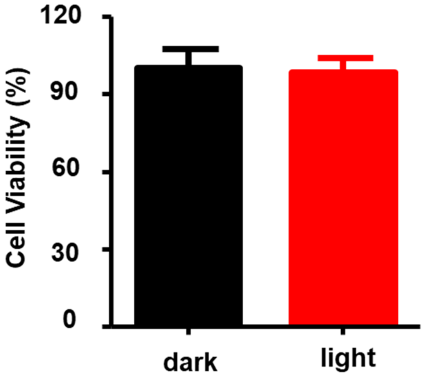


**Figure S3.** Cell survival curves of A549/CDDP after 48 h under dark and light conditions alone.
